# Method to Compute the Solute–Solvent Dispersion Contribution to the Electronic Excitation Energy in Solution

**DOI:** 10.1021/acs.jctc.2c00652

**Published:** 2022-10-03

**Authors:** Claudio Amovilli, Franca Maria Floris

**Affiliations:** Dipartimento di Chimica e Chimica Industriale, Università di Pisa, Via Giuseppe Moruzzi 13, 56124Pisa, Italy

## Abstract

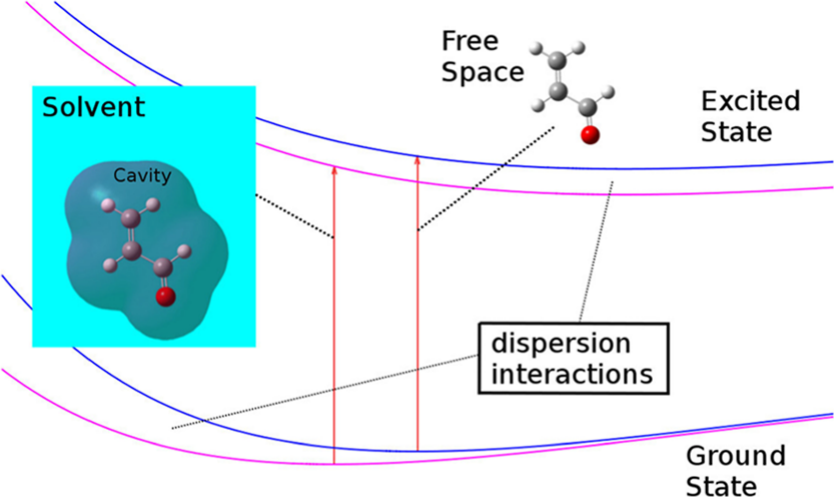

A method formulated within the polarizable continuum model of the solvent and a quantum Monte Carlo treatment of the electronic states of the solute molecule is presented for the calculation of the solute–solvent dispersion contribution to the electronic excitation energy in solution. Variational quantum Monte Carlo is exploited to measure the fluctuations of the electronic electric field of the solute molecule to compute the London’s dispersion forces with the solvent. The method previously applied to the ground state of the solute is here extended to excited states. To perform the Casimir–Polder integration, we introduce a positive parameter Ω whose value is properly chosen for this purpose. We derive a general expression that for Ω = 0 reduces to that already proposed for the ground state. For an excited state, Ω must be less than the first transition electronic energy of the solvent molecule but greater than the transition energy from the ground to excited electronic state of the solute molecule. Benchmark calculations were performed on the n → π* transition for formaldehyde, acrolein, and acetone in six solvents, including water, ethanol, cyclohexane, chloroform, carbon tetrachloride, and toluene, and the π → π* transition of acrolein in cyclohexane. Solvents are characterized by their ionization potential and the refractive index at frequency Ω. In all cases, we found that the dispersion solute–solvent interaction stabilizes the excited state of the solutes leading to red (negative) solvatochromic shifts.

## 1 Introduction

The motion of the electrons of interacting systems, such as atoms or molecules, is mutually correlated by the respective instantaneous fluctuations of the charge density linked to the motion of the electrons themselves. For the dimer in the ground state, these effects produce forces of an always attractive nature between the systems in question. A clear interpretation of these was given by London in 1930.^1^ The nature of these weak forces is exclusively quantum, and their treatment, from a computational point of view, requires very accurate methods which go beyond the medium field approximation. From a formal point of view, the dispersion energy, that is, the interaction energy due to such fluctuations, is a small contribution to the dynamic electronic correlation energy.^2^

In complex systems, this interaction is present for every pair of molecules and therefore should be considered in the study of processes involving a system of interest, molecule, or aggregate in a complicated environment. Since this treatment is already relatively heavy on a pair of isolated molecules, it is rather difficult to include it in calculations on complex systems. For this reason, simplified approaches are generally used.

The most widely used approach to include these types of interactions is based on techniques that refer to dispersion corrected energy density functionals. Following these methodologies, the dispersion energy is added by an atom–atom damped *R*^–6^ potential in which the atom–atom *C*_6_ coefficients are directly related to atomic properties.^3,4^ The damping function is introduced to avoid short-range singularities.

One of the most popular approaches of this kind is given by Tkatchenko and Scheffler.^5^ In this case, the *C*_6_ coefficients are formulated in terms of homonuclear parameters and atomic static polarizabilities. Such parameters and properties are then explicitly written in terms of the electronic density and, for this reason, appropriate contributions are derived to include dispersion interactions in the quantum mechanical (QM) Hamiltonian. On this basis, density functional theory (DFT) and time-dependent DFT (TDDFT) calculations, which include dispersion, are then achievable at a pure QM level. For the cases in which one of the two interacting parts, namely, the complex environment, needs to be treated at a lower level of description, some modification of the Tkatchenko and Scheffler approach has been proposed to perform the computation at a QM/molecular mechanical (MM) level.^6,7^

In a further simplification of the description of the environment, a continuum model can be used. In this type of calculation, the system of interest, to be studied at a QM level, is placed in a cavity of a given volume and shape created in the continuum medium representing the environment. Among these kind of approaches, it is worth mentioning that the method introduced by Marenich et al.^8^ combines the static polarizability of the solute and the solvent refractive index and can be applied also to excited states. In a more refined polarizable continuum model (PCM), the medium is responsible for the so-called reaction field acting on the system of interest and enters the QM Hamiltonian. A very thorough PCM treatment, which includes a significant part of the solute–solvent dispersion interaction, was recently proposed by Guido et al.^9^ The method is based on the Open Quantum System Theory. In this theory, the system of interest treated at the quantum level is immersed in a polarizable medium (the bath). In this context, the environment responds both in a delayed way and through its fluctuations to the polarization of the system. The authors, in this work, have developed a specific time-dependent Schrödinger equation, thus also obtaining information on the electronic states of the system of interest. The dispersion contribution is contained in the model, but the stochastic term accounting for solvent fluctuations is missing.^9^

In the past, a reliable reaction field for dispersion interactions was introduced by Amovilli and Mennucci for the process of solvation.^10^ This approach has been implemented subsequently in a TDDFT context by Cupellini et al.^11^ in order to extend the applicability to electronic vertical excitations.

Many recent developments have in common the extension to TDDFT of a model of dispersion interactions which comes essentially from a ground-state theory. When one of the interacting systems is in an excited state, also de-excitations contribute to the sum that gives the total dispersion energy. The effect of these contributions cannot be accounted for by any treatment that has a ground-state reference. In the present study, we want to explore this problem by developing a model which is formulated for a system in which one molecule is in an excited state. As a first step, we start from a continuum model, leaving to a further work the generalization to a possible discrete model.

In a previous work,^12^ we have presented a method to estimate the dispersion interaction energy between two molecules based on the measure of the electronic electric field fluctuations by means of quantum Monte Carlo (QMC) methodologies. The approach has been extended to the calculation of the dispersion contribution to the free energy of solvation within a continuum model framework. An explicit expression has been given, and test calculations have been performed on atomic solutes in water as a solvent. Here, we show for the first time the generalization of the method to nonspherical solutes in ground and low-lying excited states and in various solvents. The method involves the accurate calculation of the electronic wave function of the solute in ground and excited states, while the solvent is treated as a continuum and is characterized by the refractive index and the ionization potential. We present results for different cavities. In all our calculations, we observe a red shift due to this contribution in the vertical electronic excitation energy of the solute.

The paper is organized as follows: we start by reviewing our previous continuum model and we illustrate the modification to implement the calculation for electronic vertical excitations of solutes. Next, we illustrate in detail some calculations on a set of test examples, and we discuss the results. Finally, we draw the conclusions with suggestion for future directions.

## 2 Theory

Starting from London’s interpretation of intermolecular dispersion forces,^1^ in our previous work,^12^ we have introduced a measure of the electronic electric field fluctuations that can be used to evaluate the strength of such interactions. In our approach, we distinguish the two interacting systems: one, A say, is the system of interest, to be studied at a high level of the theory, and the other, B say, is treated as a probe and is modeled in terms of its dipolar polarizability α_B_. Because we started from London asymptotic formula, in our paper,^12^ A and B were atoms.

Our main achievement has been the following formula for the computation of interatomic dispersion energy
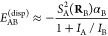
1where *I*_A_ and *I*_B_ are the ionization potentials of A and B, and for any system A of interest
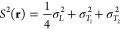
2in which σ^2^ is the fluctuation of the electronic electric field along a given direction, namely
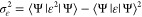
3*L* is the direction along **r** and *T*_1_ and *T*_2_ are perpendicular to **r**. In order to extend the validity of the above expression of *E*^(disp)^ to finite, eventually short, interatomic distances, the electric field is damped by a function *f*_2_ as follows^12^
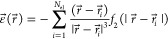
4where
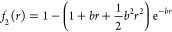
5in which *b* is a parameter characteristic of the system A.

We used QMC to find the wavefunction Ψ of the system A, and we computed the electric field fluctuations from the sampled electronic configurations.

In a second step, always in our paper,^12^ we extended the dispersion energy equation to the calculation of the relevant contribution to the solvation free energy for a solute A in a solvent B. Solvent polarization was introduced within a PCM framework by means of the Clausius–Mossotti equation. The resulting equation for the dispersion contribution to the solvation free energy is
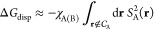
6where
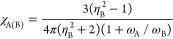
7in which η_B_ is the solvent (B) refractive index, ω_A_ and ω_B_ are the characteristic London’s formula frequencies of solute (A) and solvent, and *C*_A_ is the solute cavity.

In our previous paper, we applied eq 6 on atomic solutes in aqueous solution, but this equation is more general and is valid also for a polyatomic solute A of any shape, the quantity *S*_A_^2^ being related to the electronic electric field fluctuations. However, eq 6 is restricted to solutes in the ground state. In fact, eq 6 is the final result of a mathematical development which involves the Casimir–Polder type integration^13^ and the Unsold’s approximation.^14^ The Casimir–Polder type integral is the crucial point. In order to understand how to modify the equations to follow the same procedure for the excited states, we have to go back to the definition of dispersion energy between two electronic systems.

From the second-order perturbation theory and neglecting the overlap, the usual definition of dispersion energy between two molecules A and B in the states, respectively, a and b is^2^
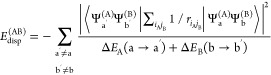
8where the sum runs over all possible simultaneous excitations/de-excitations in both molecules. If a and b are the two ground states, all transition energies are positive. In this case, we can use the Casimir–Polder type integral^13^
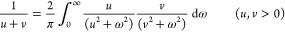
9and by putting *u* = Δ*E*_A_(0 → a^′^) and *v* = Δ*E*_B_(0 → b^′^), one obtains the relation^15,16^
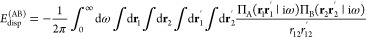
10in terms of the individual generalized frequency-dependent polarizabilities^2,15^ (X = A, B)
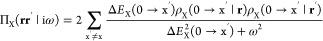
11Here, ρ_X_(0 → x′|**r**) is the transition density between the ground state and the excited state x^′2^.

In the asymptotic regime, when the distance *R*_AB_ between the two molecules is very large, eq 10 behaves as
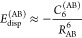
12giving for the Van der Waals coefficient *C*_6_^(AB)^, the expression
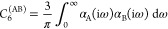
13where the frequency-dependent dipole polarizabilities α_X_(iω) come from the multipolar expansion of the above transition densities. When the frequency is imaginary, like in this case, the dipole polarizabilities are monotonically decreasing with ω, starting from the maximum value at ω = 0 and reaching zero at infinity. A typical approximation, in terms of the static polarizability α_X_(0) and the ionization potential *I*_X_, is the following^17^
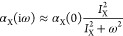
14which leads to the London’s formula^1^ for the van der Waals coefficient *C*_6_^(AB)^, namely
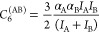
15

London’s formula has been the starting point of our previous work^12^ in which, by using the Unsold’s approximation to rewrite the static dipole polarizability, we have been able to introduce, in the expression of the dispersion energy for a given system interacting with a point model atom, the electronic electric field fluctuations of eq 3.

Let us consider at this point the case of a molecule, say A, in an excited state “a” interacting with the other molecule B in the ground state. As we will show below, we must impose the constraint
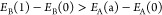
16Namely, the de-excitation of A cannot excite B. As a consequence, the dispersion interaction remains attractive.^18^

In order to perform the Casimir–Polder type integration, we take a positive number Ω such that
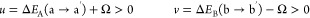
17where clearly
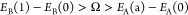
18

Then, by applying the same procedure followed for the two molecules in the ground state, the van der Waals coefficient *C*_6_^(AB)^ should be rewritten as
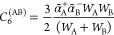
19where (see e.g., refs (18) and (19))
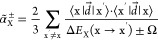
20are modified dipole polarizabilities. Here
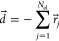
21is the usual electronic dipole operator. In eq 20, the ionization potentials have been replaced by two new parameters *W*_A_ and *W*_B_ as a result of the summation over the states of A and B. In the present work, we make use of the most intuitive assumption
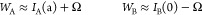
22in which the two ionization potentials are shifted by the quantity ±Ω.

At this point, we proceed, as in the previous case, with the Unsold’s approximation on  by writing
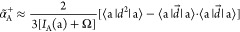
23where “a” now indicates an excited state of molecule A.

For the molecule B, we prefer to rewrite  in the following form
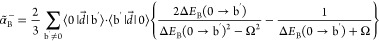
24

Although Ω is set smaller than the first transition energy of B, the first term inside the bracket could be large near resonance (Ω close to Δ*E*_B_(0 → 1)), so we apply Unsold’s approximation only to the second term. This leads to
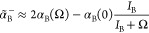
25where we regain the definitions of the static dipole polarizability α_B_(0) and of dynamic dipole polarizability α_B_(Ω) at real frequency Ω.^17^

After a few straightforward steps of algebra, one finally gets
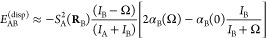
26

It is important to remark that for Ω = 0 and A in the ground state, this equation reduces to that of our previous work.

Having defined the expression of dispersion energy in terms of electric field fluctuations of the target molecule (A) and of the dipole polarizability of the surrounding molecule (B), we propose a generalization of our previous expression for the dispersion free energy of solvation. This is still in the form of eq 6, namely
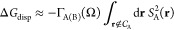
27where the new prefactor Γ_A(B)_(Ω) is now
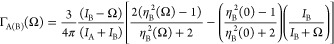
28in which we assume that the Clausius–Mossotti equation can be applied on both α_B_(0) and α_B_(Ω).

## 3 Computational Details

We performed test calculations on three solutes, formaldehyde, acrolein, and acetone. In all cases, we have studied the vertical electronic transition between the ground state and the n → π* excited state at the equilibrium geometry of the ground state. For acrolein, we studied also the transition to the π → π* excited state.

The solutes under study have been treated at the QMC level in order to generate a large number of electronic configurations to compute the integrand function *S*_A_^2^(**r**) of eq 27.

**Figure 1:**
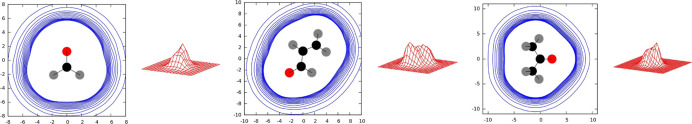
Contour plot of the function *S*^2^(**r**) of eq 27 for HCHO (left), acrolein (center), and acetone (right). The origin of the reference frame corresponds to the electronic center of charge of the solute.

In Figure 1 the contour plot of this function for the three solutes, as calculated from QMC, is displayed.

We used variational QMC (VMC) to optimize the wavefunction taken in a Slater–Jastrow form. The Jastrow factor is that of Filippi and Umrigar^20^ and included e–n and e–e two-body terms and e–e–n three-body terms. For the determinantal part of the wavefunction derived from a CASSCF setup, more precisely, we used CASSCF(4,3) for acetone and formaldehyde and CASSCF(6,5) for acrolein in order to include in the active space the π valence shell and a σ lone pair on oxygen. Here, we used the Burkatzki, Filippi, and Dolg pseudopotentials with its valence triple-zeta (VTZ) Gaussian basis set.^21^ All QMC calculations have been performed with the program CHAMP.^22^

In Table 1 we report the parameter *b* used for the calculation of the electric field fluctuations and the ionization potential evaluated at the diffusion Monte Carlo (DMC) level for the solute in vacuo in the ground and excited states. In our previous article,^12^ the parameter *b* was defined in terms of an empirical relationship that links it to an atomic hard sphere radius. In this paper, where we have molecules instead of atoms, we have extended the definition by considering the radius of equal hard spheres centered on the heavy atoms (C or O). For a single atom, the definition of such a radius leaves a fraction of 0.5 electrons outside of the sphere.^12^ In the case of molecules, on the other hand, the fraction of electrons that escapes is equal to a maximum of 0.5 electrons multiplied by the number of spheres that can be reduced in order to consider the possible overlapping of the spheres.

**Table 1:** *b* Parameter of the Damping Function *f*(*r*) (See Text) and the DMC Ionization Potential *I* (Hartree) for the Three Solutes Studied in This Work in Their Ground and n → π* Excited States

solute	state	*b*	*I*
HCHO	GS	1.67	0.4043
	n → π*	1.67	0.2503
acrolein	GS	1.60	0.3776
	n → π*	1.60	0.2349
acetone	GS	1.62	0.3634
	n → π*	1.61	0.1908

In this work, we calculated the ionization potential at the DMC level because it is accurate for this purpose, although other methods can be used alternatively, including experimental ones.

In order to illustrate the present approach, we consider six solvents that do not absorb in the same region of the n → π* transition of the above solutes. These are water and ethanol, which can form hydrogen bonds with the three solutes, cyclohexane, as an example of nonpolar solvent, chloroform, carbon tetrachloride, and toluene which are nonpolar but show a higher polarizability. According to eq 18 which establishes the range of possible values of the parameter Ω, we decided to fix the interval by comparing the vertical excitation energy of the three solutes with the simulated UV absorption spectra of the solvents computed with Gaussian^23^ with TDDFT at the B3LYP/cc-pVTZ level of the theory.

**Figure 2:**
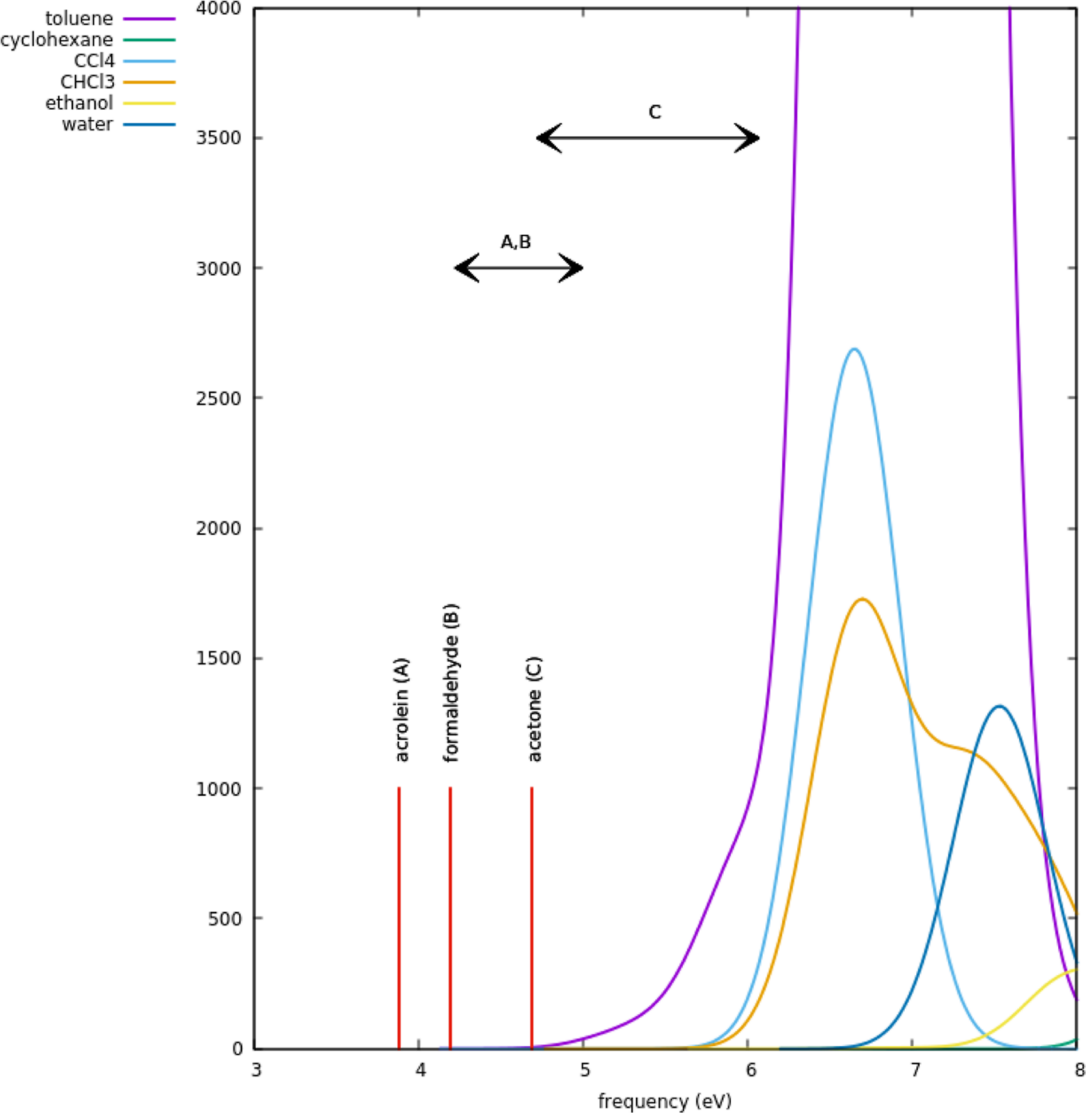
Comparison between the vertical excitation energy (DMC) of solutes and the simulated UV absorption spectra of solvents obtained from TDDFT calculations performed at the B3LYP/cc-pVTZ level of the theory. The horizontal arrows indicate the interval of Ω values (see text) used in the computation of dispersion energy contribution to the solvation free energy. The letters A, B, and C refer to the solutes.

In Figure 2 we show this comparison and display the intervals of Ω considered in this work. To compute the dispersion energy contribution, we need to evaluate the refractive index of the solvent at frequency Ω, which is in the range of ultraviolet light. For the frequency-dependent refractive index, we have used the following formula^24^
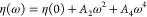
29where η(0) is the static value and *A*_2_ and *A*_4_ are the fitting parameters to reproduce the available literature data for the six aforementioned solvents. Literature data are taken from Foss and Schellman^25^ for cyclohexane, chloroform, and carbon tetrachloride from Daimon and Masumura^26^ for water and from Kozma et al.^24^ for ethanol and toluene. The fitting parameters are reported in Table 2. In the same table, we also report the experimental ionization potentials.^27^

**Table 2:** Fitting Parameters for the Frequency (eV)-Dependent Refractive Index of the Solvent Considered in This Work^a^

solvent	η(0)	*A*_2_	*A*_4_	*I*
water	1.32315	0.00201432	6.21176 × 10^–6^	0.4638
ethanol	1.35059	0.00226353	7.02316 × 10^–6^	0.385133
cyclohexane	1.41142	0.00259217	1.25986 × 10^–5^	0.3631
chloroform	1.43108	0.00260712	3.60209 × 10^–5^	0.41784
carbon tetrachloride	1.44277	0.00332168	1.48334 × 10^–5^	0.4215
toluene	1.48437	0.0016524	0.000241204	0.3245

a The last column refers to experimental ionization potentials (Hartree).

The solute molecules treated in this work are also characterized by the π → π* transition, but only for acrolein in cyclohexane can we apply the present approach. Indeed, in all other cases, it is not possible to define an appropriate value for Ω to satisfy the constraint given by eq 18. For this reason, we provide additional results for the π → π* transition of acrolein in cyclohexane.

### 3.1 Cavity

The cavity is modeled on a set of interlocking spheres centered on the solute nuclei. The radius of each sphere is a sum of two radii representing the contact of two spheres belonging to the solute and the solvent and depending on the kind of contact. For the solute counterpart, we take the atomic radius corresponding to *R*_1/2_ of Amovilli and McWeeny,^28^ namely, 3.26 *a*_0_ for carbon, 2.81 *a*_0_ for oxygen, and 2.33 *a*_0_ for hydrogen. For the solvent counterpart, instead, the construction depends strongly on the solvent. For water, we add the radius of solvent oxygen to the solute carbon and hydrogen radii, while for the solute oxygen, we add that of the water hydrogen in order to consider the hydrogen bond. Again, these two solvent atomic radii have been taken from the work of Amovilli and McWeeny^28^ and are 2.96 *a*_0_ for O and 1.86 *a*_0_ for H. For ethanol, we maintain the same approach for the sphere defined on the solute oxygen by adding the radius of hydrogen with the same value used for water; meanwhile, for the other atoms, we add a radius which represents a spherical model for the CH_3_ group and has the value of 3.932 *a*_0_. For all other solvents, we have taken a unique solvent sphere. More precisely, we consider 5.20 *a*_0_ for cyclohexane, 4.82 *a*_0_ for chloroform, 5.20 *a*_0_ for carbon tetrachloride, and 5.01 *a*_0_ for toluene. The resulting radii are displayed in Table 3.

**Table 3:** Cavity Radii (bohr) of the Interlocking Spheres Centered on Solute Nuclei

solvent	R(C)	R(O)	R(H)
water	6.22	4.67	5.29
ethanol	7.19	4.67	6.26
cyclohexane	8.46	8.01	7.53
chloroform	8.08	7.63	7.15
carbon tetrachloride	8.46	8.01	7.53
toluene	8.27	7.82	7.34

In this work, we have studied the effect of scaling the cavity on Δ*G*_disp_ of eq 27. For this purpose, we have scaled all cavity radii except for water as solvent, for which the scaling is applied only to the sphere centered on the oxygen nucleus of the solute molecule.

## 4 Results

The main purpose of this work is the evaluation of the contribution due to solute–solvent dispersion energy to the solvatochromic shift in the vertical electronic n → π* excitation for the chosen systems.

**Figure 3:**
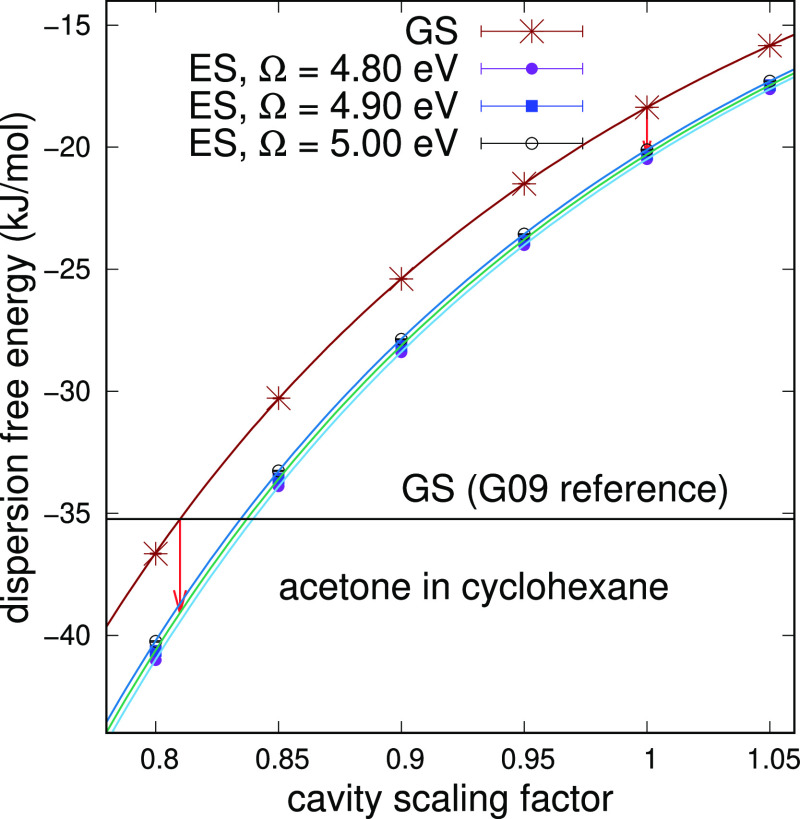
Dispersion free energy of solvation of acetone in cyclohexane for the ground and n → π* excited states, computed at different values of Ω (eV) as a function of the cavity scaling factor. The two vertical arrows display the solvatochromic shift starting from the Gaussian reference and without scaling the cavity.

**Figure 4:**
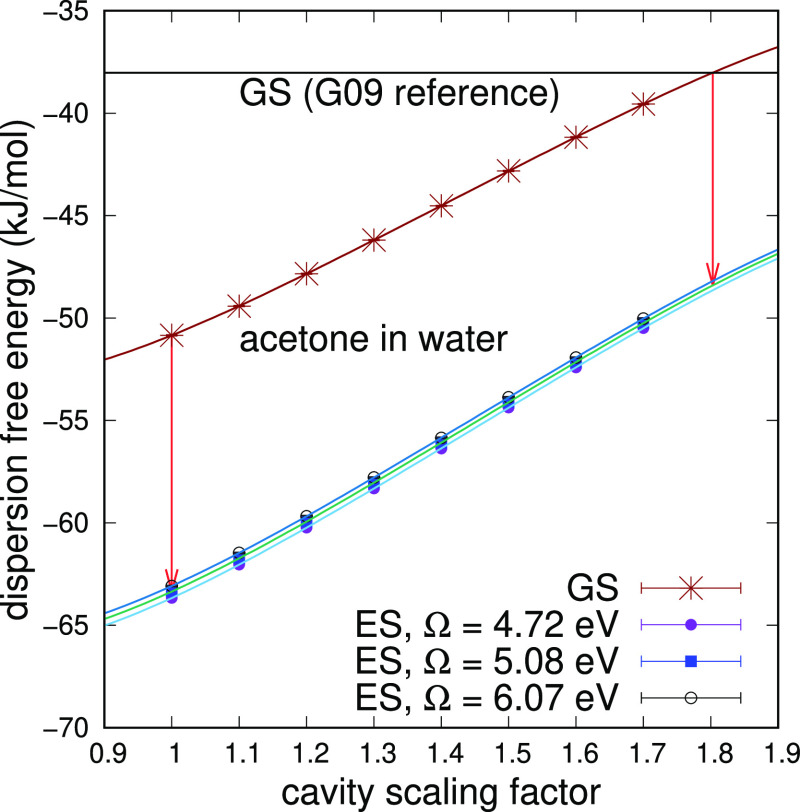
Dispersion free energy of solvation of acetone in water for the ground and n → π* excited states, computed at different values of Ω (eV), as a function of the cavity scaling factor. The two vertical arrows display the solvatochromic shift starting from the Gaussian reference and without scaling the cavity.

For all solutes and solvents, we plotted the dispersion energy contribution to the solvation free energy by varying the cavity scaling factor for ground and n → π* excited states of the solute. For the excited-state calculations, we explored various choices of Ω according to the restrictions fixed in this work. Qualitatively, all computed curves look very similar, and the effect of variation of Ω is relatively small if compared to the solvatochromic shift. The shift is always negative (red shift). Although ground- and excited-state curves look almost parallel, for comparison purposes, we decided to read the shift by fixing the scaling factor at which the ground-state dispersion energy contribution is exactly the same as that calculated by Gaussian code^23^ using its default for the solute–solvent pair. This default corresponds to a calculation using the internal parameters of the given solvent with the cavity fixed by the code (universal force field atomic radii scaled by 1.100 factor). The dispersion energy contribution in this case derives from the model of Floris and Tomasi.^29^ In all plots, we report also the shift calculated with the scaling factor equal to 1 (see Table 3). By way of example, we show here the case of acetone in cyclohexane and in water; see Figures 3 and 4. All other cases can be found in the Supporting Information. In Tables 4 and 5 are collected all estimated solvatochromic shifts. The first table refers to the Gaussian reference while the latter to the cavity with no scaling.

**Table 4:** Computed Solvatochromic Shift (eV) due to Solute–Solvent Dispersion Interaction Using the Gaussian 09 Reference for the Ground-State Dispersion Free Energy Contribution^a^

solvent	HCHO	CH_2_CHCHO	Me_2_CO
vacuo	4.19	3.88	4.69
water	–0.051(1)	–0.076(1)	–0.108(2)
ethanol	–0.020(4)	–0.03(1)	–0.052(2)
cyclohexane	–0.021(2)	–0.026(3)	–0.040(3)
chloroform	–0.031(2)	–0.044(2)	–0.068(1)
carbon tetrachloride	–0.033(2)	–0.050(3)	–0.072(1)
toluene	–0.031(2)	–0.0436(5)	–0.075(1)

a The row vacuo refers to the calculated vertical excitation energy (eV).

**Table 5:** Computed Solvatochromic Shift (eV) Due to Solute–Solvent Dispersion Interaction without Scaling the Cavity Radii^a^

solvent	HCHO	CH_2_CHCHO	Me_2_CO
vacuo	4.19	3.88	4.69
water	–0.052(1)	–0.075(7)	–0.130(3)
ethanol	–0.023(1)	–0.026(7)	–0.047(3)
cyclohexane	–0.0096(9)	–0.014(2)	–0.020(2)
chloroform	–0.024(1)	–0.038(1)	–0.056(1)
carbon tetrachloride	–0.019(1)	–0.032(1)	–0.052(1)
toluene	–0.016(1)	–0.024(1)	–0.043(1)

a The row vacuo refers to the calculated vertical excitation energy (eV).

The red (negative) shifts in all cases confirm that the solute–solvent dispersion interaction stabilizes the n → π* excited state probably due to the fact that such a state is more polarizable than the ground state. The magnitude of the shift, if compared to the vertical transition energy, is about 1–2 percent, namely, some 5 nm in a typical UV spectrum. The solvent water gives the greatest shift. In our model, the design of the cavity takes into account the possibility of the formation of a hydrogen bond. In such cases, dispersion interactions are augmented due to the shortening of the distances. All other solvents give instead about one half of the water shift with little differences among themselves. As already said, the change in the scale of the cavity does not change too much the shift in the range of factors considered in this work. This can be seen by comparing the data of Tables 4 and 5. Nevertheless, we suggest to use the Gaussian reference for this calculation. In the two aforementioned tables, the number in parentheses refers as usual to the uncertainty in the last digit. This quantity depends on the interval of Ω but does not affect too much the reliability of the average reported shift data.

Our results appear to be in agreement with the literature data. First of all, it must be said that the experimental solvatochromic shifts include all effects of solute–solvent interactions and, in the case of protic solvents such as water, for this type of solute, the shift is dominated by the electrostatic interactions. It is well known, in fact, that electrostatic interactions are responsible for a relatively strong blue shift to the n → π* transition, for the three solutes considered here in water, due to H-bond. The known experimental data (see, e.g., ref (30)) have been substantially confirmed by theoretical calculations in which electrostatics was explicitly included at both QM/MM^30,31^ and PCM levels.^32−34^ Furthermore, we remark that, in such cases, even the solute–solvent Pauli repulsion plays a not negligible role by lowering in part the electrostatic blue shift (see, e.g., refs (35) and (36)). A similar behavior was found for other systems with the same kind of transition in water solution.^6,37^ However, Guareschi et al.^38^ made calculations on a cluster of acrolein with 11 water molecules. In this work, they performed both VMC and DMC calculations, finding for the vertical n → π* transition energy 4.40(2) eV with VMC and 4.30(2) eV with DMC. The difference between DMC and VMC, namely −0.10(2) eV, could be ascribed to dispersion interactions, considering that DMC includes certainly this contribution as opposed to VMC which could lack this quantity. Both our data of Tables 4 and 5 are in agreement with this possible estimate of the solute–solvent dispersion contribution for acrolein in water.

For nonprotic solvents, acetone in cyclohexane represents a system in which dispersion interactions could dominate the solvatochromic shift. In this case, the experimental solvatochromic shift can be used in this work for direct comparison purposes. For this system, Renge^39^ found a shift of −400 cm^–1^. From Table 4, we obtain −323 cm^–1^ and, from Table 5, −162 cm^–1^. Both the results are in line with the literature and, in particular, the first from Table 4 is in good agreement.

For acetone and acrolein in cyclohexane, we can make a comparison with also the theoretical work of Cupellini et al.^11^ In Table S3 of the Supporting Information provided by the authors, the dispersion–repulsion contribution of the excitation energy shift is reported at different values of a parameter *c*_s_. This parameter has been introduced to modulate the dispersion interaction effects in the PCM response matrix in their TDDFT approach. They performed the calculation at the M062X/6-311+G(2d,2p) level. The data of the table strongly depend on the value of *c*_s_. The authors have suggested that an optimal value of *c*_s_ should be obtained by comparison with experimental data when available. They also state that a value of *c*_s_ > 1 is not unexpected because of de-excitations, as they have shown by using a simplified model. Moreover, the maximum value of *c*_s_ according to this model should be 2. For acetone and acrolein in cyclohexane, the contribution of Pauli repulsion should be less than 1 percent of the reported values, considering the general analysis presented in their study. If we assume all dispersion, we recover for *c*_s_ the values of 1.69 for acetone and 1.85 for acrolein by taking the results of Table 4. This comparison shows that the two sets of results are consistent, despite the differences of the two approaches and the empirical nature of the parameter *c*_s_.

**Figure 5:**
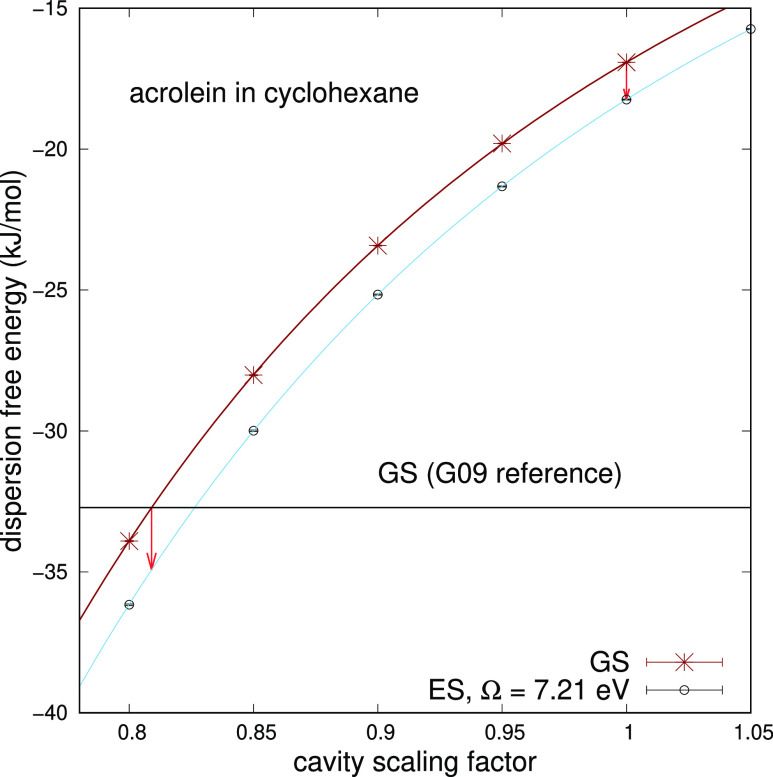
Dispersion free energy of solvation of acrolein in cyclohexane for the ground and π → π* excited states, computed at Ω equal to 7.21 eV (172 nm), as a function of the cavity scaling factor. The two vertical arrows display the solvatochromic shift starting from the Gaussian reference and without scaling the cavity.

For acrolein in cyclohexane, we have also studied the π → π* transition. At the DMC level, the electronic vertical transition energy found is 6.93 eV (179 nm or 55,900 cm^–1^). The cyclohexane starts to absorb significantly at about 60,000 cm^–1^ (7.44 eV or 167 nm)^40^ (see also Figure 2). On the basis of constraint on Ω (see eq 18), we have chosen the value of 7.21 eV (172 nm or 58,140 cm^–1^). For this value of Ω we extrapolate a refractive index η of 1.60 by using the fitting function
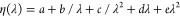
30where λ is given in nm and the data are taken from the work of Foss and Schellman.^25^ The source data are provided in a range of wavelength far from 172 nm, and therefore our extrapolation should be taken with care. Nevertheless, the value of 1.60 can be reasonably used for the estimate of Δ*G*_disp_. Dispersion free energy contribution results are plotted in Figure 5. Without scaling the cavity, the computed solvatochromic shift contribution is −0.0081(2) eV while at the Gaussian 09 reference is −0.0120(4) eV. For both cavities, the shift is about half of the values found for the n → π* transition. Even for this transition, we can make a comparison with the work of Cupellini et al.^11^ If we take their Table S3, and considering now a Pauli repulsion contribution of 0.0414 eV as reported in their Table S4, our Gaussian 09 reference should give a dispersion–repulsion contribution of 0.0185 eV, which is consistent with a *c*_s_ value of 1.75.

Finally, it is important to note that in our calculations, we used the wave function of the isolated molecule. We worked as in a standard perturbation approach in which the computed term is a correction to a given order. The other solute–solvent interactions here are omitted, but their effect reflects in a relatively small perturbation of the solute wave function. The variation on the dispersion contribution will result in a correction to a higher order. We are confident that this correction is very small. For this kind of system, Cupellini et al.^11^ found the quasi additivity of electrostatic and nonelectrostatic shifts, which supports our assumption in this work. A first possible improvement could be the use of the perturbed wave function, if available, in the calculation of the electronic electric field fluctuations, our approach being not limited to the use of an isolated molecule wave function. In a more general procedure, since the Δ*G*_disp_ of this work is a functional of the wave function, it should in principle be possible to improve the wave function of the solute coherently with the dispersion interactions included. In the current study, we developed a parallel code to evaluate the integral of eq 27 in order to calculate the contribution of the dispersion free energy. A single calculation takes minutes on our cluster but, if this operation has to be repeated several times in the process of optimization, the method could become prohibitive. For this reason, it is necessary to develop appropriate algorithms and significantly modify the QMC code. This will be an object for future study.

## 5 Conclusions

In this paper, a general method for calculating the solvatochromic shift due to dispersion interactions is presented. The method is formulated within the PCM of the solvent and can be applied to any solute that exhibits a vertical electronic transition energy lower than the transition energy of the solvent to its first excited state. The peculiarity of this approach lies precisely in a special treatment for considering the contribution of the de-excitations of the solute in the calculation of the dispersion energy of the solute–solvent complex system. Here, we extend to excited states our previous approach for solutes in the ground state.^12^ The main achievement of the present study is embodied in eqs 27 and 28. As for the ground state, the integral outside the solute cavity of eq 27 is based on the calculation of the electronic electric field fluctuations of the solute molecule via the function *S*^2^(**r**) of eq 2 now evaluated for the excited state of interest. The prefactor of eq 28 takes instead a new more general form depending on a special parameter, namely Ω, here introduced to perform Casimir–Polder integration to achieve the final expression of eq 27. Ω is an energy and takes a value between the vertical transition energy of the solute and the vertical transition energy of the solvent to its first excited state. For a solute in the ground state, Ω is 0 and the prefactor gives back the expression of our previous paper.^12^ Even in this work, we use QMC to compute electric field fluctuations from the solute electronic wave function.

We tested the method for the n → π* transition for the three carbonylic compounds formaldehyde, acetaldehyde, and acetone in six different solvents ranging from water to toluene. We evaluated the shift on the transition energy due to dispersion under different conditions related to the cavity size and to the choice of the parameter Ω. We have observed a relatively modest dependence on these conditions. This is a positive result considering that there is always some ambiguity in the definition of the cavity and in the choice of Ω. In all cases, we found a negative (red) shift which is consistent with the idea that the excited state is more polarizable than the ground state, at least for n → π* transitions. Water as a solvent gives the greatest effect reflecting the fact that, due to H-bond which leads to a closer contact, solute–solvent dispersion interactions are bigger in this case. The solvatochromic shift is relatively small at the limit of detectability being of the order of about 5 nm in the UV region.

In the additional test performed on the π → π* transition of acrolein in cyclohexane, we have found a similar solvatochromic shift but about half that of the n → π* case.

As a conclusion, we can say that the approach presented in this work is able to capture the small effect due to dispersion energy on the value of the vertical electronic transition energy for a solute dispersed in a continuum solvent. The method requests the explicit wavefunction for the ground and excited states of the solute, and in principle, eq 27 can be used to define an appropriate term in the solute QM Hamiltonian. If the effect is of the magnitude found in this work, we do not expect significant changes in the wave function. For the future, we will explore this possibility in order to extend the method to a self-consistent procedure to take into account precisely the reaction field of the solvent in the QM Hamiltonian.

Another very interesting aspect is that of reformulating the approach in a discrete treatment of the environment. In order to do this, one has to go back to eq 26. This equation should be reviewed to be adapted to a molecular aggregate and reparameterized. This will be the object of a future work.

## Supporting Information Available

Plots of the dispersion free energy of solvation for ground and excited states for the three solutes in the six different solvents considered in this work against the cavity size and different choices of the parameter Ω (PDF)
