# Supplementary material for: Method to Compute the Solute–Solvent Dispersion Contribution to the Electronic Excitation Energy in Solution
Source: J Chem Theory Comput. 2022 Oct 3;18(11):6816–25. doi: 10.1021/acs.jctc.2c00652 (PMC9648189; doi:10.1021/acs.jctc.2c00652)
Supplement: Supplementary file 1 — ct2c00652_si_001.pdf [file ct2c00652_si_001.pdf]

Supporting information for: A method to compute the solute-solvent dispersion contribution to the electronic excitation energy in solution

Claudio Amovilli* and Franca Maria Floris*

Dipartimento di Chimica e Chimica Industriale, Università di Pisa, Via Giuseppe Moruzzi 13, 56124 Pisa,Italy

E-mail: claudio.amovilli@unipi.it; francamaria.floris@unipi.it

*To whom correspondence should be addressed

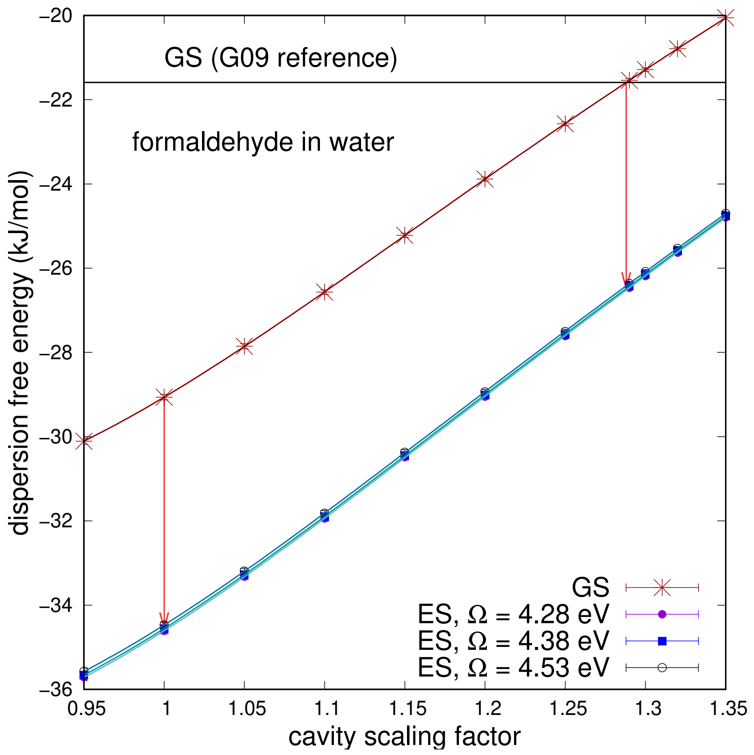

Figure S1: Dispersion free energy of solvation of formaldehyde in water for the ground and $n \rightarrow \pi^*$ excited states, computed at different values of Ω (eV), as a function of the cavity scaling factor. The two vertical arrows display the solvatochromic shift starting from the Gaussian reference and without scaling the cavity.

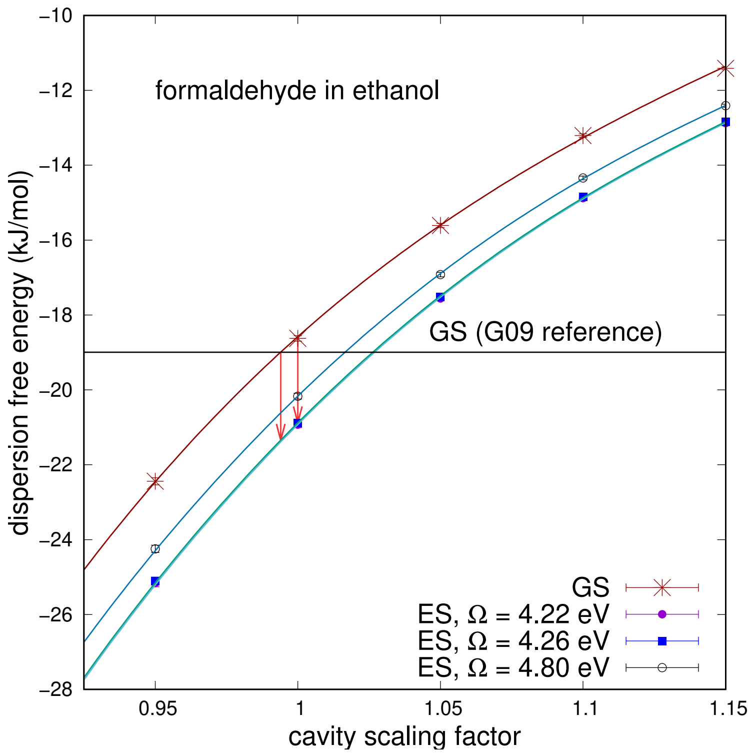

Figure S2: Dispersion free energy of solvation of formaldehyde in ethanol for the ground and $n \rightarrow \pi^*$ excited states, computed at different values of Ω (eV), as a function of the cavity scaling factor. The two vertical arrows display the solvatochromic shift starting from the Gaussian reference and without scaling the cavity.

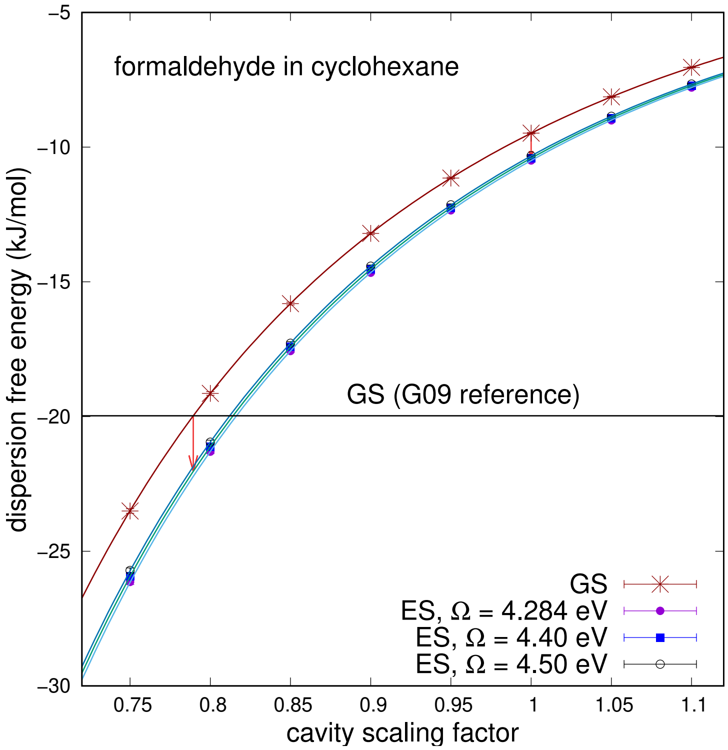

Figure S3: Dispersion free energy of solvation of formaldehyde in cyclohexane for the ground and $n \rightarrow \pi^*$ excited states, computed at different values of Ω (eV), as a function of the cavity scaling factor. The two vertical arrows display the solvatochromic shift starting from the Gaussian reference and without scaling the cavity.

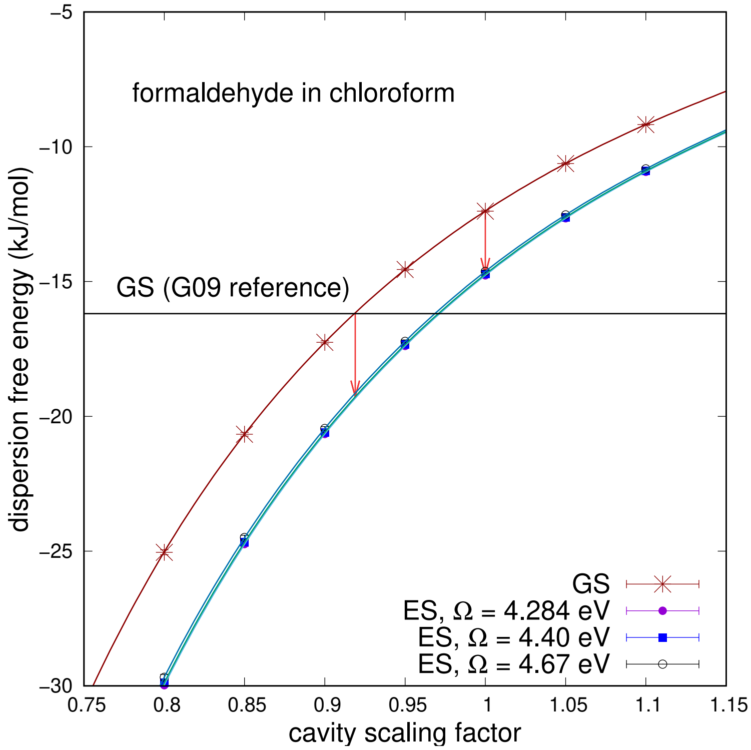

Figure S4: Dispersion free energy of solvation of formaldehyde in chloroform for the ground and $n \rightarrow \pi^*$ excited states, computed at different values of Ω (eV), as a function of the cavity scaling factor. The two vertical arrows display the solvatochromic shift starting from the Gaussian reference and without scaling the cavity.

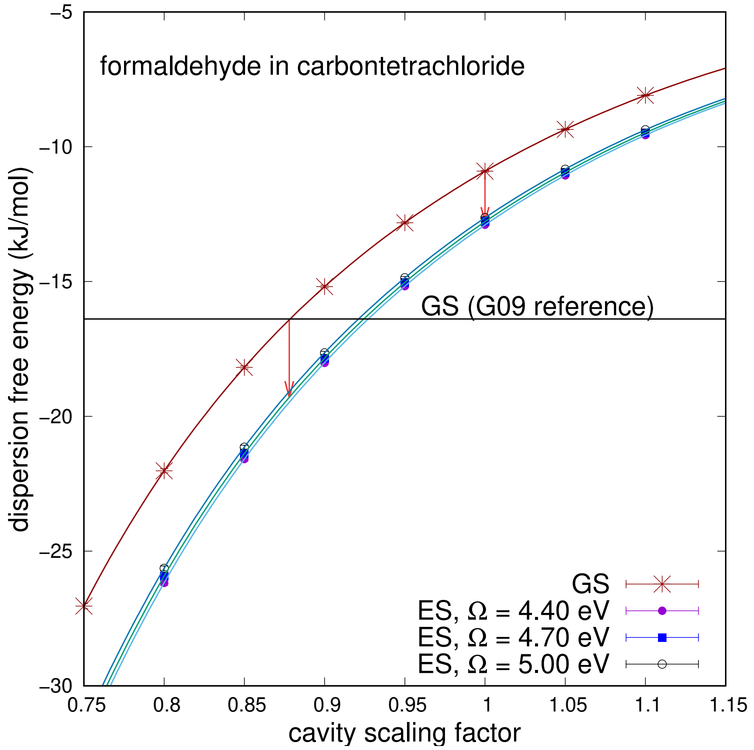

Figure S5: Dispersion free energy of solvation of formaldehyde in carbontetrachloride for the ground and $n \rightarrow \pi^*$ excited states, computed at different values of Ω (eV), as a function of the cavity scaling factor. The two vertical arrows display the solvatochromic shift starting from the Gaussian reference and without scaling the cavity.

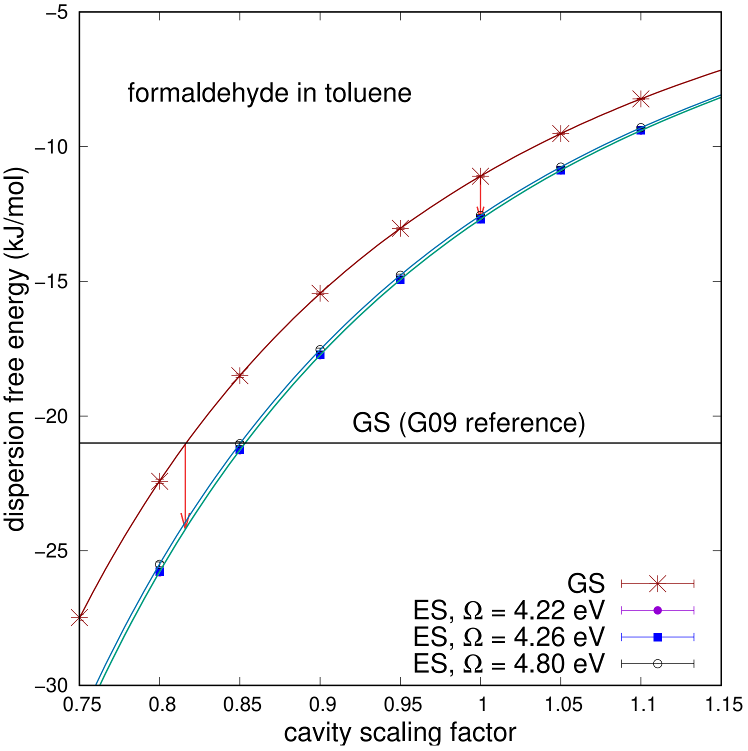

Figure S6: Dispersion free energy of solvation of formaldehyde in carbontetrachloride for the ground and $n \rightarrow \pi^*$ excited states, computed at different values of Ω (eV), as a function of the cavity scaling factor. The two vertical arrows display the solvatochromic shift starting from the Gaussian reference and without scaling the cavity.

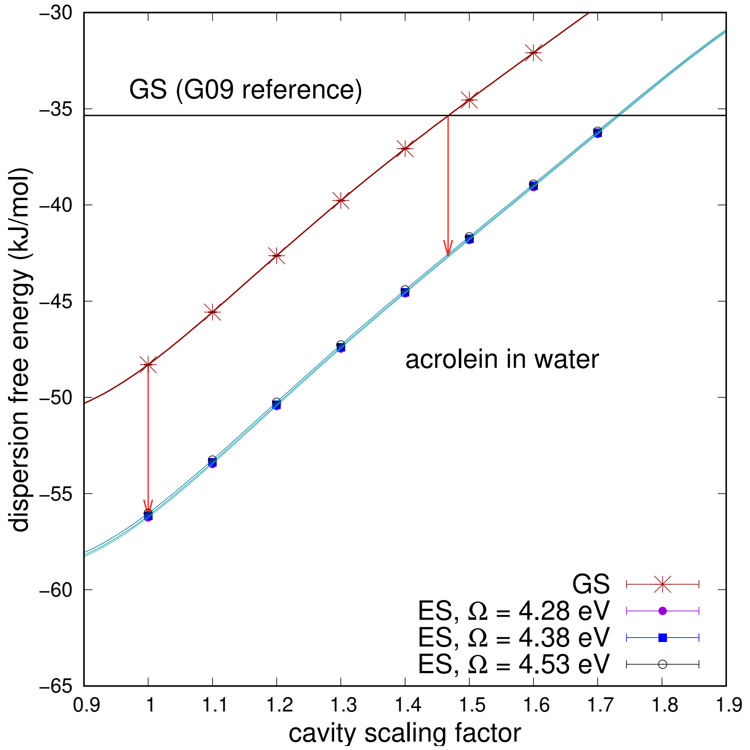

Figure S7: Dispersion free energy of solvation of acrolein in water for the ground and $n \rightarrow \pi^*$ excited states, computed at different values of Ω (eV), as a function of the cavity scaling factor. The two vertical arrows display the solvatochromic shift starting from the Gaussian reference and without scaling the cavity.

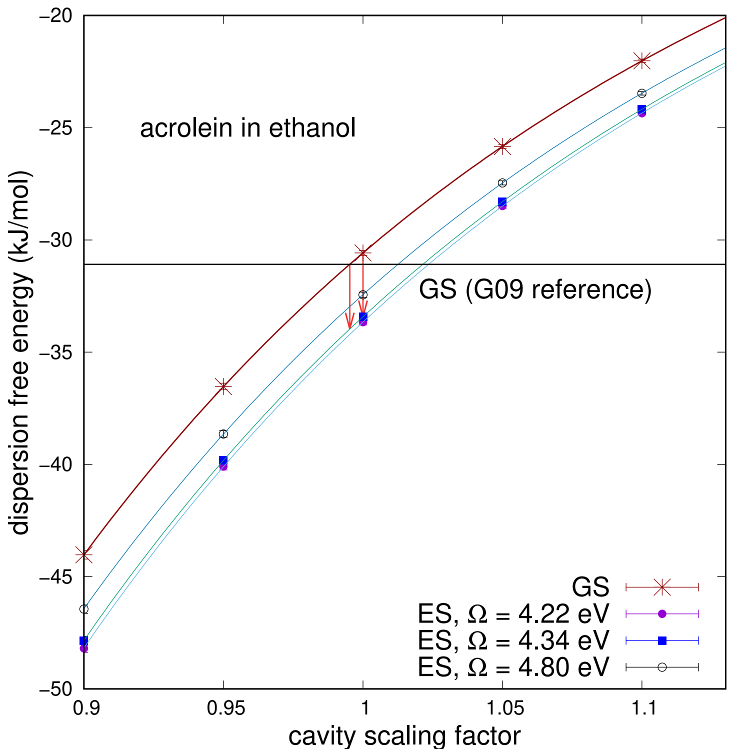

Figure S8: Dispersion free energy of solvation of acrolein in ethanol for the ground and $n \rightarrow \pi^*$ excited states, computed at different values of Ω (eV), as a function of the cavity scaling factor. The two vertical arrows display the solvatochromic shift starting from the Gaussian reference and without scaling the cavity.

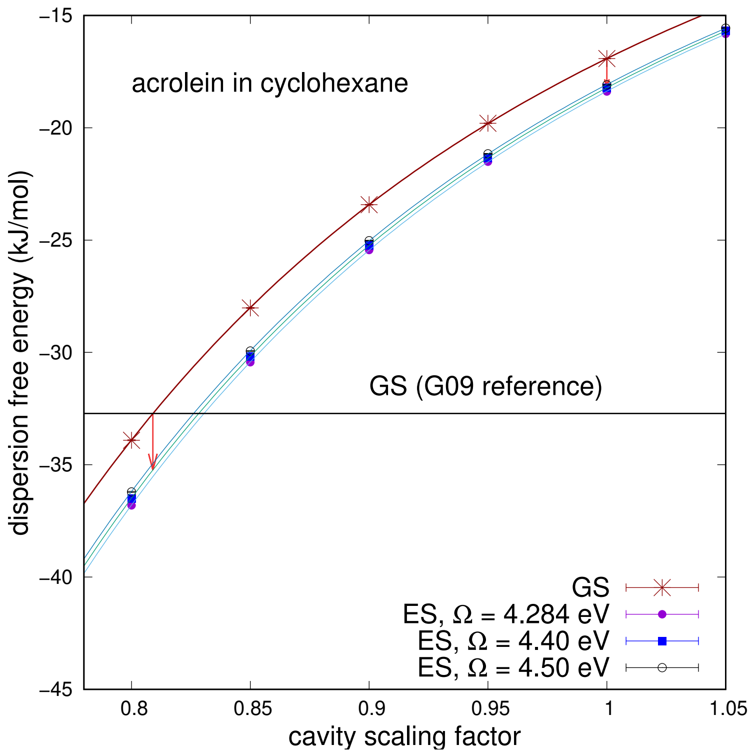

Figure S9: Dispersion free energy of solvation of acrolein in cyclohexane for the ground and $n \rightarrow \pi^*$ excited states, computed at different values of Ω (eV), as a function of the cavity scaling factor. The two vertical arrows display the solvatochromic shift starting from the Gaussian reference and without scaling the cavity.

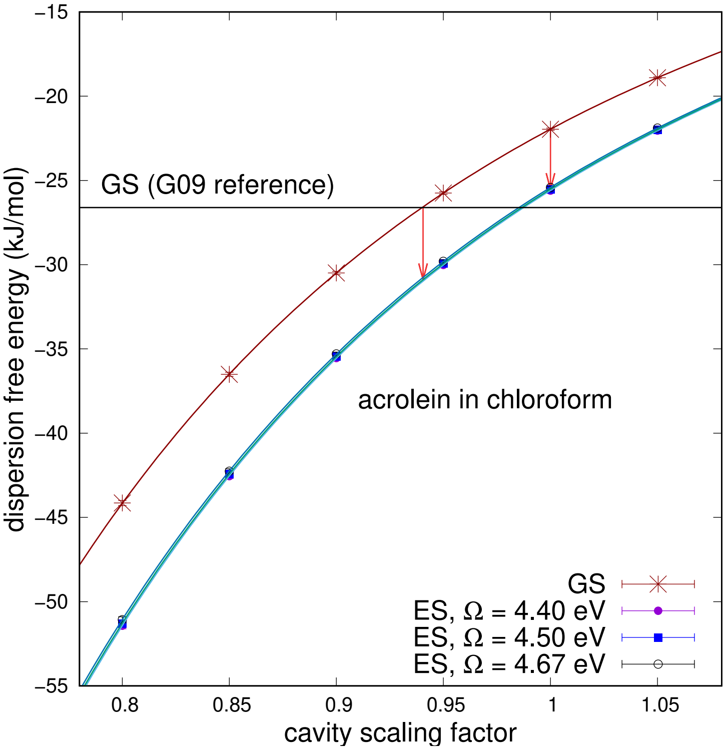

Figure S10: Dispersion free energy of solvation of acrolein in chloroform for the ground and $n \rightarrow \pi^*$ excited states, computed at different values of Ω (eV), as a function of the cavity scaling factor. The two vertical arrows display the solvatochromic shift starting from the Gaussian reference and without scaling the cavity.

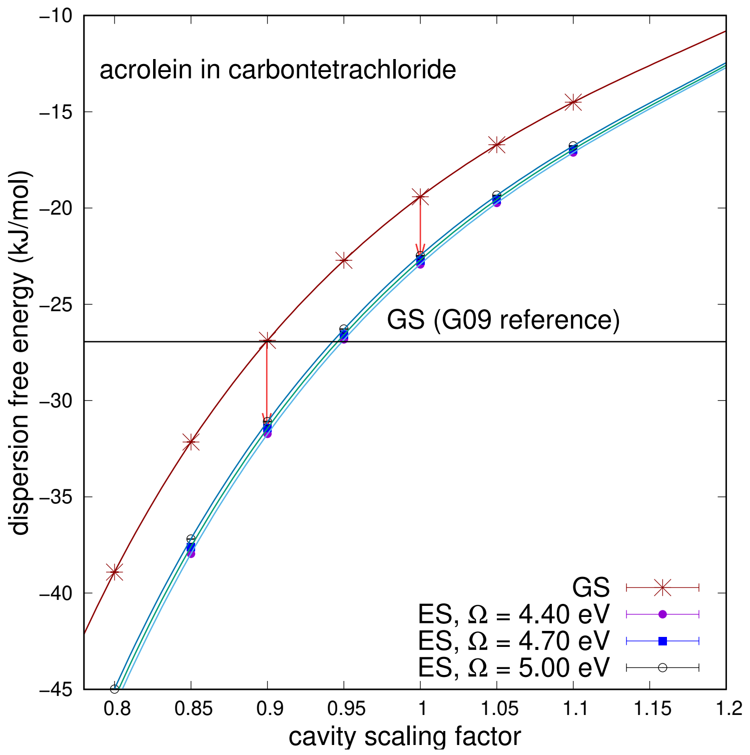

Figure S11: Dispersion free energy of solvation of acrolein in carbontetrachloride for the ground and $n \to \pi^*$ excited states, computed at different values of Ω (eV), as a function of the cavity scaling factor. The two vertical arrows display the solvatochromic shift starting from the Gaussian reference and without scaling the cavity.

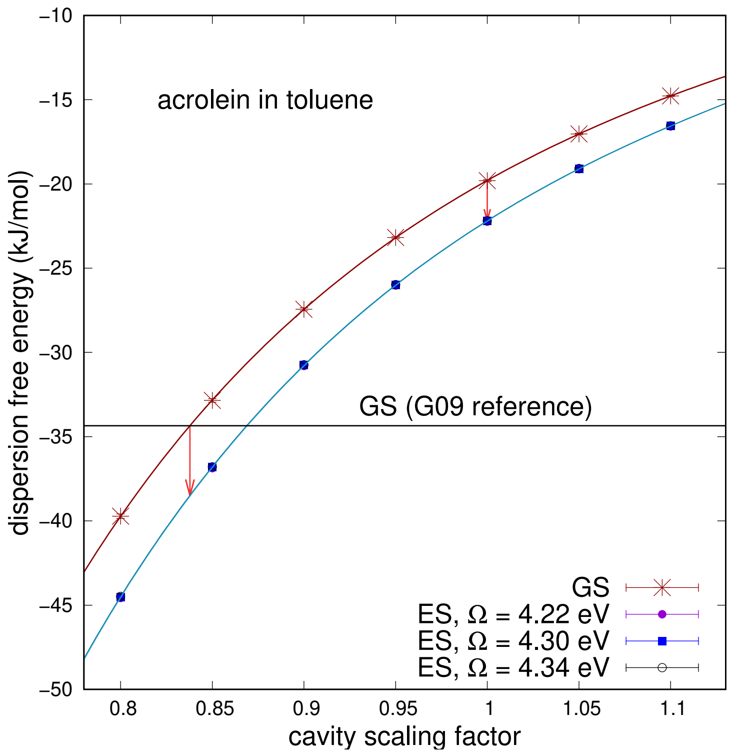

Figure S12: Dispersion free energy of solvation of acrolein in toluene for the ground and $n \rightarrow \pi^*$ excited states, computed at different values of Ω (eV), as a function of the cavity scaling factor. The two vertical arrows display the solvatochromic shift starting from the Gaussian reference and without scaling the cavity.

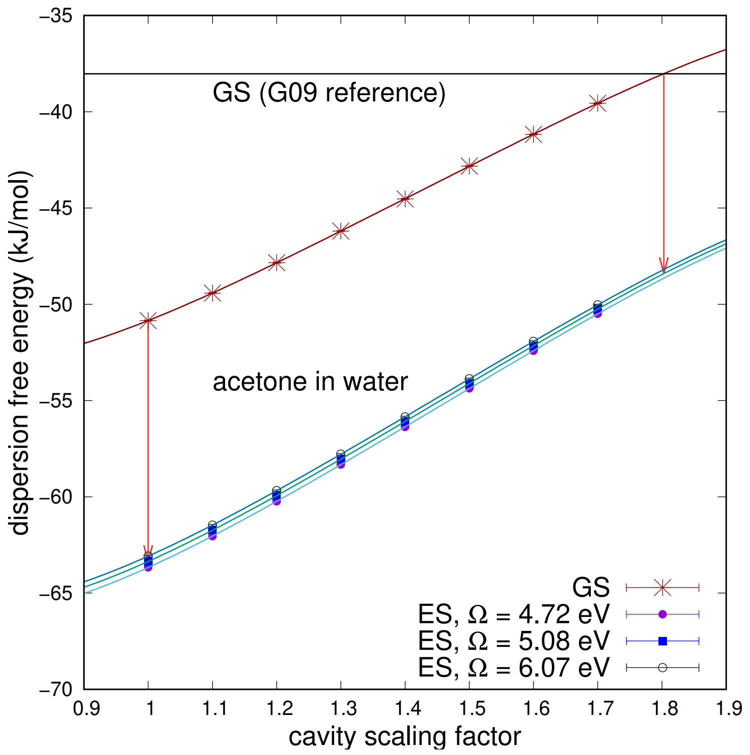

Figure S13: Dispersion free energy of solvation of acetone in water for the ground and $n \to \pi^*$ excited states, computed at different values of Ω (eV), as a function of the cavity scaling factor. The two vertical arrows display the solvatochromic shift starting from the Gaussian reference and without scaling the cavity.

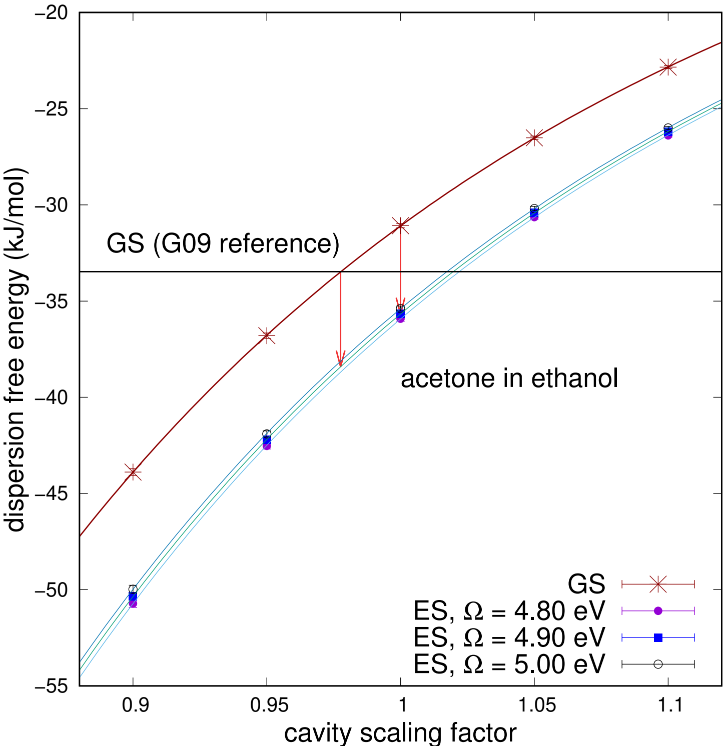

Figure S14: Dispersion free energy of solvation of acetone in ethanol for the ground and $n \rightarrow \pi^*$ excited states, computed at different values of Ω (eV), as a function of the cavity scaling factor. The two vertical arrows display the solvatochromic shift starting from the Gaussian reference and without scaling the cavity.

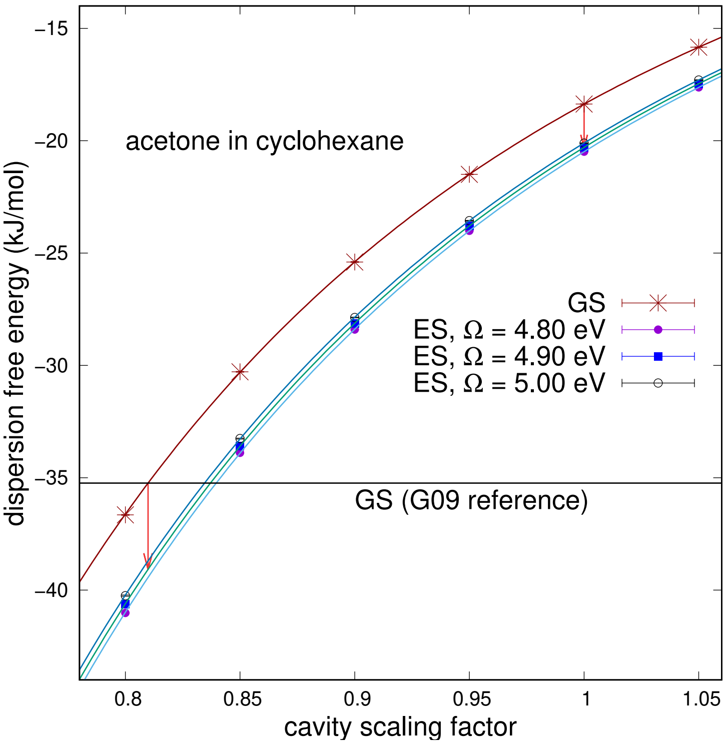

Figure S15: Dispersion free energy of solvation of acetone in cyclohexane for the ground and $n \rightarrow \pi^*$ excited states, computed at different values of Ω (eV), as a function of the cavity scaling factor. The two vertical arrows display the solvatochromic shift starting from the Gaussian reference and without scaling the cavity.

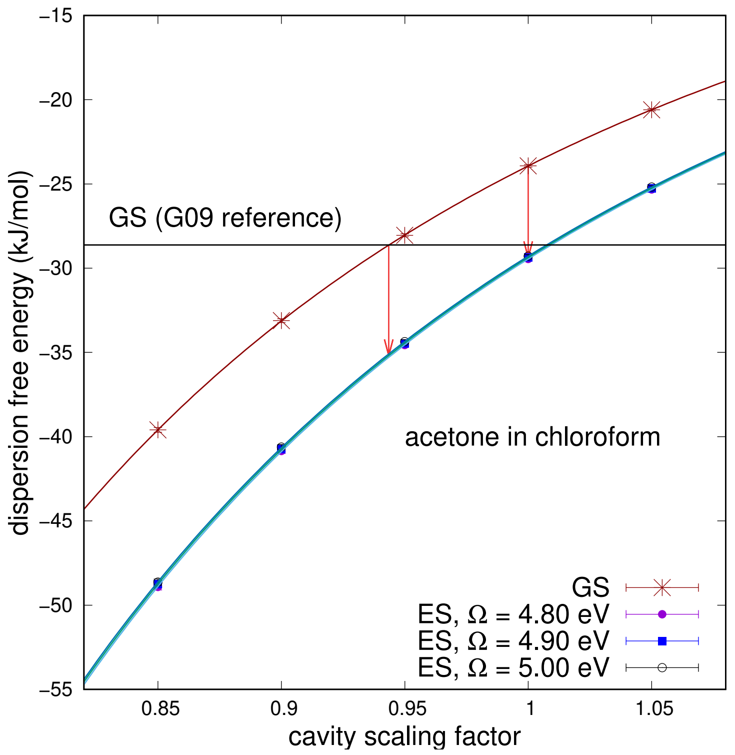

Figure S16: Dispersion free energy of solvation of acetone in chloroform for the ground and $n \rightarrow \pi^*$ excited states, computed at different values of Ω (eV), as a function of the cavity scaling factor. The two vertical arrows display the solvatochromic shift starting from the Gaussian reference and without scaling the cavity.

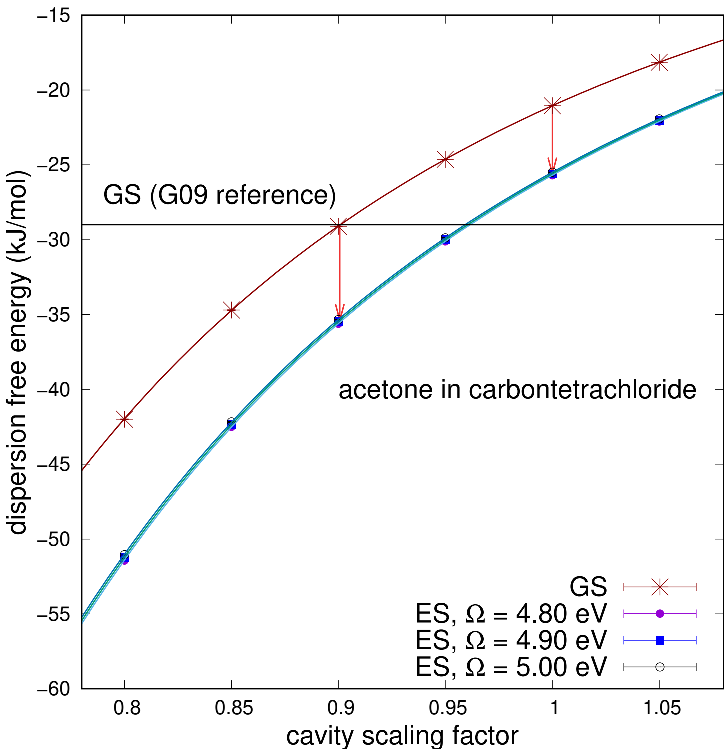

Figure S17: Dispersion free energy of solvation of acetone in carbontetrachloride for the ground and $n \rightarrow \pi^*$ excited states, computed at different values of Ω (eV), as a function of the cavity scaling factor. The two vertical arrows display the solvatochromic shift starting from the Gaussian reference and without scaling the cavity.

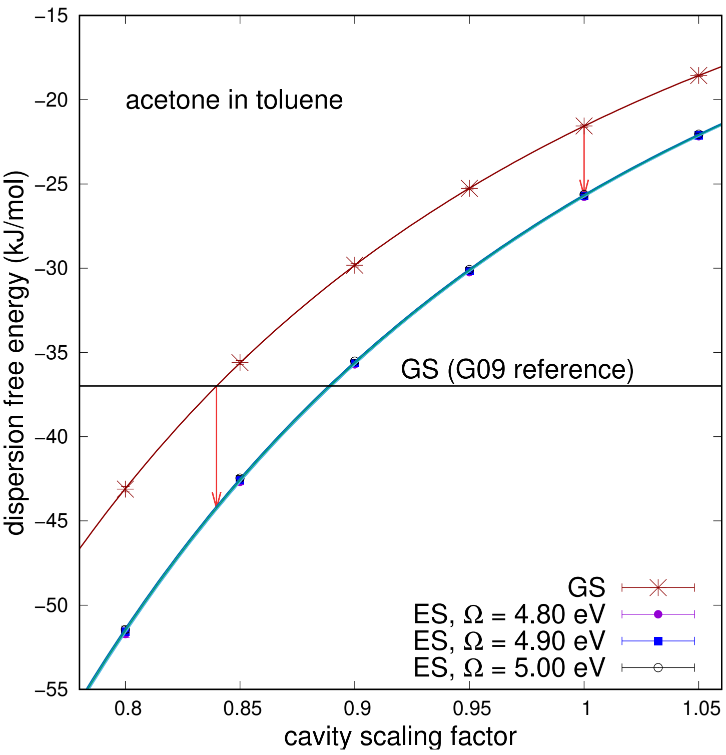

Figure S18: Dispersion free energy of solvation of acetone in toluene for the ground and $n \rightarrow \pi^*$ excited states, computed at different values of Ω (eV), as a function of the cavity scaling factor. The two vertical arrows display the solvatochromic shift starting from the Gaussian reference and without scaling the cavity.
